# Transcranial pulsed electromagnetic fields for multiple chemical sensitivity: study protocol for a randomized, double-blind, placebo-controlled trial

**DOI:** 10.1186/1745-6215-14-256

**Published:** 2013-08-16

**Authors:** Marie Thi Dao Tran, Sine Skovbjerg, Lars Arendt-Nielsen, Karl Bang Christensen, Jesper Elberling

**Affiliations:** 1 The Danish Research Centre for Chemical Sensitivities, Department of Dermato-Allergology, Copenhagen University Hospital Gentofte, Ledreborg Allé 40, 2. th., DK-2820 Gentofte, Denmark; 2 Center for Sensory–Motor Interaction, Department of Health Science and Technology, Aalborg University, Frederik Bajers Vej 7 D3, DK-9220 Aalborg, Denmark; 3 Section of Biostatistics, Department of Public Health, University of Copenhagen, Øster Farimagsgade 5, DK-1014 Copenhagen K, Denmark

**Keywords:** Multiple chemical sensitivity, Pulsed electromagnetic fields, Re5 therapy, Re5 independent system, Randomized controlled trial

## Abstract

**Background:**

Multiple chemical sensitivity (MCS) is a chronic condition of unknown etiology. MCS is characterized by recurrent nonspecific symptoms from multiple organ systems in response to chemical exposures in concentrations that are normally tolerated by the majority of the population. The symptoms may have severe impact on patients’ lives, but an evidence-based treatment for the condition is nonexisting. The pathophysiology is unclarified, but several indicators point towards abnormal processing of sensory signals in the central nervous system. Pulsed electromagnetic fields (PEMF) offer a promising new treatment for refractory depression and can be targeted at the brain, thereby activating biochemical cell processes.

**Methods/Design:**

In a parallel, randomized, double-blind, placebo-controlled trial conducted at the Danish Research Centre for Chemical Sensitivities, the effects of PEMF in MCS patients will be assessed using the Re5 Independent System. Based on sample size estimation, 40 participants will be randomized to either PEMF therapy or placebo. The allocation sequence will be generated by computer. All involved parties (that is, participants, investigators, the research nurse, and the statistician) will be blinded to group allocation. The participants will receive PEMF therapy or placebo applied transcranially 30 minutes twice a day for 7 days a week over 6 consecutive weeks. Outcomes will be measured at baseline, once weekly during treatment, post treatment, and at 2.5-month and 4.5-month follow-up according to a predefined timetable. The primary outcome will be a measurement of the impact of MCS on everyday life. The secondary outcomes will be measurements of MCS symptoms, psychological distress (stress, anxiety or depressive symptoms), capsaicin-induced secondary punctate hyperalgesia, immunological markers in serum, and quality of life.

**Discussion:**

This trial will assess the effects of PEMF therapy for MCS. Currently, there is no treatment with a documented effect on MCS, and in terms of healthcare there is very little to offer these patients. There is thus a great need for well-conducted randomized trials aimed at assessing possible treatment effects. A positive outcome will pave the way for improved healthcare and understanding of this very disabling and overlooked condition.

**Trial registration:**

ClinicalTrials.gov, NCT01834781

## Background

Multiple chemical sensitivity (MCS) is a chronic condition of unknown etiology [1-3]. MCS symptoms are triggered by exposure to common odors and other airborne chemicals in low concentrations; for example, perfume, freshly printed newspapers or magazines, cooking fumes or new electronic equipment. Symptoms related to the central nervous system (CNS; for example, headache, exhaustion, and concentration difficulties) are commonly reported, but other organ systems such as the airways, muscles, and joints are also often involved in symptom reports [4]. In severe cases, MCS can lead to loss of employment and social isolation [2,5,6]. Other labels have been ascribed to the symptoms (for example, chemical intolerance and idiopathic environmental intolerance) but MCS is widely used in the scientific literature and will be used here without reference to any assumptions about etiology. The etiology and pathophysiology of MCS remain largely unclarified. However, current findings suggest that both biological and psychological mechanisms are involved [4,7-10]. Within the biological spectrum, the theory of an abnormal response in the CNS termed central sensitization is receiving increasing scientific attention [7-9,11], partly because this hypothesis is compatible with the polysymptomatic manifestations from multiple organ systems and with the often reported association with symptoms of negative affectivity and stress [12-17]. The theory of central sensitization is further supported by recent findings. During odor provocations, brain imaging studies have demonstrated a significantly reduced activity in the cerebral areas that process olfactory stimuli in MCS patients compared with healthy controls [18,19]. Suggestions have been made that the observed reduction in cerebral activity reflects reduced activity in the inhibitory brain circuits, thus resulting in an increased response to normal sensory input [19]; that is, central sensitization. In controlled experimental pain studies, the presence of central sensitization in MCS has also been supported by findings of enlarged areas of capsaicin-induced secondary mechanical hyperalgesia [20,21]. Capsaicin (the active component in chili peppers) injections in the skin induce secondary mechanical hyperalgesia, which is an increased sensitivity to mechanical stimuli in the skin surrounding the injection site and is considered to be a CNS response.

The development of MCS has been described in two or three stages [22]: an initiating chemical exposure induces sensory irritation, and is followed by an elicitation phase in which symptoms are elicited by chemicals in nontoxic concentrations. As a possible explanation, it has been hypothesized that the initial chemical exposure triggers plastic changes in the CNS, which result in a changed response to a specific chemosensory input – analogous to the development of a persisting pain condition following an acute pain incident [8]. A third, generalization phase has been described in which symptom-eliciting odors and chemicals begin to involve other and unrelated chemical exposures [2]. This phase is also compatible with the localization of MCS in the CNS. Whether the immune system plays a part is unknown, but increased levels of immunological markers have been demonstrated among MCS patients [23].

Very few intervention studies aimed at MCS have been published [24,25] and, apart from these, there have only been a few published case reports. Hence, it is not currently possible to make evidence-based therapeutic recommendations for MCS [26]. In accordance with the CNS hypothesis, a transient but yet pronounced effect of electroconvulsive therapy has been reported in a few MCS patients [27]. As electroconvulsive therapy involves general anesthesia and muscle relaxants in advance of cerebral electrostimulation and also potential transient amnesia, it is a less attractive theraputic option for MCS. Transcranial magnetic stimulation and pulsed electromagnetic fields (PEMF) are more modern technologies to achieve electrostimulation of the brain without employing anesthesia or muscle relaxants. Both technologies involve the generation of a time-varying magnetic field, which consists of magnetic flux lines. A change in the magnetic field produces an electrical current in conductive materials (for example, tissue). PEMF differ from transcranial magnetic stimulation in mainly two aspects: PEMF are based on weak magnetic fields and are therefore completely unable to induce action potentials in excitable cells; and the field change alternates rapidly between minimum and maximum [28]. The electrical currents generated by PEMF are thus weak and lead to activation of the biochemical processes in the cells, especially the growth-related responses.

In 1977 the first therapeutic use of PEMF took place and was used to treat pseudoarthrosis and nonunions [29]. Animal studies since then have shown that PEMF can be used to enhance peripheral nerve regeneration [30-33], activate neural tissue [34], promote wound healing [35], enhance the proliferation of chondrocytes [36], and promote angiogenesis [37] and vasodilation [33,38]. Currently, PEMF are still mostly used for the promotion of bone growth and have been approved by the US Food and Drug Administration to promote the healing of certain fractures [39]. However, the scope of application in humans is expanding, because PEMF have also been shown to have beneficial effects on osteoarthritis [40,41] as well as antidepressive effects when applied to refractory depressions [42]. The substantial individual and societal costs of MCS and the lack of evidence-based therapeutic options call for well-conducted randomized trials evaluating the effect of a possible therapeutic option such as PEMF. In the forthcoming study, PEMF will be applied transcranially to treat MCS in a randomized, double-blind, placebo-controlled trial.

### Objectives

The main purpose of the forthcoming study is to investigate whether PEMF application using the Re5 Independent System (Re5 Aps, Frederiksberg, Denmark) can reduce the impact of MCS on everyday life, such as the ability to perform domestic chores, take part in social activities, attend work, and so forth. The secondary purposes are to investigate whether the intervention can decrease MCS symptoms, psychological distress (stress, anxiety or depressive symptoms), capsaicin-induced secondary punctate hyperalgesia, immunological markers in serum and increase quality of life.

### Hypotheses

The primary hypothesis is that PEMF therapy can significantly reduce the impact of MCS on everyday life in the intervention group compared with the control group. The secondary hypotheses are that PEMF therapy can significantly decrease: general symptoms (that is, symptoms that are commonly experienced by MCS patients); exposure-related symptoms (that is, symptoms that are elicited by exposure to common odors and chemicals); stress, anxiety or depressive symptoms if present; and the area of capsaicin-induced secondary punctate hyperalgesia; the serum concentration of immunological markers (that is, IL-1β, IL-2, IL-4, IL-5, IL-6, IL-8, IL-10, IL-12, IL-13, IL-17, IL-21, IL-22, IL-23, TNFα, IFNγ); and can significantly improve quality of life.

## Methods/Design

The study will be conducted as a parallel, randomized, double-blind, placebo-controlled trial with follow-up after 2.5 and 4.5 months.

### Participants

Participants will be recruited among MCS patients who are registered at the Danish Research Centre for Chemical Sensitivities and have agreed to be contacted for research purposes, or MCS patients who have had recent contact with the Department of Dermato-Allergology, Copenhagen University Hospital Gentofte, Denmark.

Forty participants will be included in the period from 1 April 2013 to 26 July 2013. In the event that fewer participants are included during this period, the inclusion period will be extended to 1 November 2013, to make it possible to obtain the full number of participants.

### Inclusion criteria

All participants must be between 18 and 75 years of age and meet the extended consensus criteria for MCS [43], which will be put into practice as follows: symptom duration of at least 6 months; symptoms in response to at least two of 11 categories of chemical exposure; at least one CNS symptom and one symptom from another organ system; symptoms causing lifestyle or functional impairments that score ≥35 on the Quick Environmental Exposure and Sensitivity Inventory (QEESI) Life Impact Scale; symptoms occurring when exposed, and improving or resolving when triggering exposures are removed; and symptoms being triggered by exposure levels that do not induce symptoms in other individuals exposed to the same levels. Lastly, all participants must also provide written informed consent.

### Exclusion criteria

Participants will be excluded if they report: previous PEMF therapy; psychosis or a comparable disorder; epilepsy; cerebral tumors; leukemia or malignancies in the head or neck region; having a pacemaker or other active implants; pregnancy or nursing; unreliable contraception; drug or alcohol abuse; a pending application or intention to apply for early retirement; initiation of pharmacological treatment that has not stabilized; or participation in another research study.

### Withdrawal

Participants will be withdrawn from the study treatment if this is their wish, if they for some reason cannot complete the required PEMF treatments, or if they experience adverse effects at an unacceptable level. Withdrawn participants will be asked to fill in the weekly questionnaires until follow-up has been completed.

### Randomization

Participants will be randomly allocated with a 1:1 ratio and in block sizes of 10 to either the intervention group or the control group. The manufacturer of the Re5 device (Re5 Aps) will be responsible for the generation of the allocation sequence by computer for sequential participant numbers. Participants will be numbered sequentially according to their entry into the study. From the allocation sequence, sequentially numbered smartcards will be loaded with the allocated treatment. The allocation sequence will be kept locked up in two copies, one of which the investigators will have access to if a serious adverse event occurs.

### Blinding

Re5 devices will be identical for both study groups and will be operated using a smartcard, upon which the type and duration of treatment is loaded. Two types of smartcards will be produced: smartcards that are loaded with active treatments, and smartcards that are loaded with inactive (placebo) treatments. As the appearances of the smartcards will be identical, both participants and investigators will be blinded to treatment allocation. Due to a limited time frame, the allocation sequence will be unmasked for the investigators when the last participant has completed PEMF therapy to be able to conduct the statistical analyses on the post treatment data. The unmasking of the sequence for the participants will take place when all participants have completed the last follow-up. Since the outcome measures at follow-up are self-rated by the participants and there will be no communication of any kind between the investigators and the participants, we do not expect that the premature unmasking of the allocation sequence for the investigators will have any influence on the follow-up data. A research nurse will remain fully masked and will ensure that follow-up data are collected.

### Intervention

PEMF will be applied using the Re5 Independent System, which consists of the Re5 Independent (pulse generator), the Re5 Applicator Head (seven coils), and the Re5 Smartcard. Participants will receive PEMF therapy or placebo applied transcranially for 7 days a week over 6 consecutive weeks. The duration of treatment will be 30 minutes twice a day; that is, morning (06:00 to 09:00 hours) and evening (17:00 to 20:00 hours). Participants will receive instructions from the investigators on how to operate the Re5 device and the treatment will take place in the participants’ homes. PEMF therapy is painless and participants will be able to read or do whatever comes naturally to them while wearing the Re5 device.

Participants who receive pharmacological therapy for MCS or another condition will continue their treatment unchanged during the study. All medication use will be registered.

### Control visits/calls

During treatment, control visits will be arranged for all participants at weeks 1, 2, and 4 after initiation of treatment. At these visits, the smartcards – which contain electronic information on if and when the participants have received the scheduled treatments – will be monitored in order to check compliance. The monitoring will be performed without disclosing the concealed treatment allocation. Furthermore, the control visits will serve to monitor the outcome measurements (filling in the questionnaires) and to register adverse effects. At weeks 3 and 5 an email reminder will be sent in advance to ensure that questionnaires are completed. At this point, the participants will also be contacted by telephone to keep compliance up and if necessary once more to remind them to fill in the questionnaires. A research nurse, who is also blinded to group allocation, will be assigned to perform the control visits/calls.

### Baseline characteristics and outcome measurements

To characterize participants at baseline the following parameters will be registered: age, gender, smoking, comorbidities, medications, asthma, and neuroticism as assessed using the NEO Personality Inventory. Asthma will be assessed using questions adopted from the Stage 1 questionnaire of the European Community Respiratory Health Study [44]. Asthma will be defined in accordance with criteria employed by the European Community Respiratory Health Study as an affirmative answer to at least one of the following questions: Have you been woken by an attack of shortness of breath at any time in the last 12 months? Have you had an attack of asthma in the last 12 months? Are you currently taking any medicine (including inhalers, aerosols or tablets) for asthma?

Outcomes will be measured at baseline (week 0), once weekly during treatment (weeks 1 to 5), post treatment (week 6), and at 2.5-month and 4.5-month follow-ups as shown in Table 1. The self-administered questionnaires will be completed using an online questionnaire tool.

**Table 1:** Timetable for outcome measurements

	**Treatment**							**Follow-up**	
	**Week 0**^**a**^	**Week 1**	**Week 2**	**Week 3**	**Week 4**	**Week 5**	**Week 6**^**b**^	**2.5 months**	**4.5 months**
QEESI Life Impact	X	X	X	X	X	X	X	X	X
QEESI Symptom severity	X	X	X	X	X	X	X	X	X
QEESI Chemical intolerances	X	X	X	X	X	X	X	X	X
SDS	X	X	X	X	X	X	X	X	X
Individual tasks	X	X	X	X	X	X	X	X	X
Noise sensitivity	X						X	X	X
SCL-92 Depressive symptoms	X	X	X	X	X	X	X	X	X
SCL-92 Anxiety symptoms	X	X	X	X	X	X	X	X	X
SCL-92 Somatization	X	X	X	X	X	X	X	X	X
PSS-10	X	X	X	X	X	X	X	X	X
HAM-D_6_	X	X	X	X	X	X	X	X	X
WHOQOL-BREF	X						X		X
Capsaicin-induced secondary hyperalgesia	X						X		
Immunological markers	X						X		

^a^Baseline measurements.

^**b**^Post treatment measurements.

QEESI, Quick Environmental Exposure and Sensitivity Inventory; SDS, Sheehan Disability Scale; SCL-92, Symptom Check List-92; PSS-10, Perceived Stress Scale-10; HAM-D_6_, 6-item Hamilton Depression Rating Scale; WHOQOL-BREF, World Health Organization Quality of Life – Brief version.

#### Primary outcome

##### Quick environmental exposure and sensitivity inventory

The QEESI was developed as a screening questionnaire for MCS to facilitate the recording of patient anamneses [45]. In this study, three of five original scales will be used by means of an evaluated Danish translation [46]: Symptom Severity Scale, Chemical Intolerance Scale, and Life Impact Scale. Each scale contains 10 items that produce a score ranging from 0 to 100. The QEESI Life Impact Scale is the primary outcome measure in this trial.

#### Secondary outcomes

##### Quick environmental exposure and sensitivity inventory

The QEESI Symptom Severity and Chemical Intolerance Scales will be used to measure the severity of general symptoms and of exposure-related symptoms, respectively.

##### Sheehan disability scale

The Sheehan Disability Scale is widely used in psychiatry, but has also been applied to many other chronic illnesses. This scale uses visuospatial, numeric and descriptive anchors to measure impaired functioning in three domains: work, social life and family life [47]. The scale generates four disability scores, one for each domain and a total score by adding up the three individual domain scores.

##### Individual self-selected tasks

In addition to the Life Impact Scale used as the primary outcome measure, the participants will be asked to select three tasks in the areas of work, social life and family life, which are impaired by MCS at baseline. The degree of impairment associated with each task is scored on a scale from 0 to 10 with visuospatial, numeric, and descriptive anchors similar to the Sheehan Disability Scale.

##### Noise sensitivity

To measure the participants’ sensitivity to noise, they will be asked to grade their responses to 10 different noises (for example, drone of a machine, stroke of a hammer, rustling of paper) on a 5-point Likert scale.

##### Symptom Check List-92

The Symptom Check List-92 subscales for depression, anxiety and somatization will be included. These subscales comprise 35 items on which responses are rated on a 5-point Likert scale ranging from ‘not at all’ to ‘very much’. The Symptom Check List-92 has been validated in a general Danish population and normative data have been established [48,49].

##### The 6-Item Hamilton Depression Rating Scale

The 6-item Hamilton Depression Rating Scale is a short self-administered measure of depression. The scale has been shown to be as sensitive as the more widely used 17-item Hamilton Depression Rating Scale to measure antidepressive treatment effects [50].

##### Perceived Stress Scale

The short version of the Perceived Stress Scale consists of 10 questions and measures self-perception of stress by grading how different life situations are perceived. The Perceived Stress Scale-10 has been shown to be a valid and reliable measure of perceived stress [51].

##### World Health Organization Quality of Life

The brief version of the World Health Organization Quality of Life is a short multidimensional questionnaire, which measures health-related quality of life. The scale consists of four domains: physical health, psychological well-being, social relationships and environment [52].

##### Capsaicin-induced secondary punctate hyperalgesia

Eight linear vectors will be outlined, arranged radiating at 45° angles on the volar side of the right forearm halfway between the cubital fossa and the wrist. Participants will then receive an intradermal injection of 0.1 ml capsaicin in a concentration of 3.3 μM (1 μg/ml, 0.01% solution) at the meeting point of the eight vectors using a 29-gauge disposable needle producing a circular blister with a radius of 0.5 cm. A handheld mechanical probe (diameter 0.6 mm, weight 50.1 g; Centre for Sensory-Motor Interaction, Aalborg University, Aalborg, Denmark) with a blunt tip, which exerts the same force at each application, will be used to determine the area of secondary punctate hyperalgesia at 10 and 30 minutes post injection. During the procedure, participants will be blindfolded and the probe will be applied to the skin for 1 second with an interstimulus interval of 2 seconds, starting from a point well outside the injection site and then sequentially reapplied to the skin moving along a vector towards the injection site in steps of 0.5 cm. The participants will be instructed to report when the pricking sensation changes in intensity or character to become more intense, painful, burning or otherwise different. When the participant reports a change in sensation at two successive points, the first point will be marked. This procedure will be repeated along all eight vectors (V_1_ to V_8_). The eight marks will be connected to form an area of secondary punctuate hyperalgesia. The area will be calculated using trigonometry by the rule of the area of a triangle, adding up and subtracting the area of the capsaicin blister:

$$\begin{array}{ll} \mathrm{Area}= & 0.5\cdot \sin\left(45{}^{\circ}\right)\cdot \left(V_{1}\cdot V_{2}+V_{2}\cdot V_{3}+\ldots +V_{7}\cdot V_{8}\right)\\ & \left(+V_{8}\cdot V_{1}\right)–\left({\pi \cdot 0.5}^{2}\right) \end{array}$$

An area will be calculated only when there are at least two neighboring marks. A double assessment at 10 and 30 minutes post injection will be used to derive a mean area of secondary punctate hyperalgesia. In addition, capsaicin-induced pain will be measured by means of a visual analog scale with a low anchor point (no pain) and a high anchor point (worst pain imaginable). The visual analog scale measurement will be transformed into a numerical value using a ruler.

##### Immunological markers in serum

Blood samples will be collected from all participants twice during the study: one sample at baseline and one sample post treatment. Plasma will be separated by centrifuge within 30 minutes, frozen at −80°C and stored until analyses in batches. The serum concentration of the following regulating inflammatory cytokines will be assessed: IL-1β, IL-2, IL-4, IL-5, IL-6, IL-8, IL-10, IL-12, IL-13, IL-17, IL-21, IL-22, IL-23, TNFα, IFNγ.

### Sample size

The sample size calculation for the primary outcome measure, the QEESI Life Impact Scale, is based on a two-sample *t* test. A recent study among Danish MCS patients found a mean value of 61.5 with a standard deviation of 24.3 on the Life Impact Scale [46]. In this study, the intervention is expected to induce a 40% decrease on this scale. We thus expect the mean QEESI score in the intervention group to decrease to 36.9 and the mean QEESI score in the control group to remain unchanged. Since we do not know the standard deviation of the change score we base our sample size calculation on the assumption that the standard deviation of the change score is 24.3. Under these assumptions and with a two-sided significance level set at 0.05, the study will require 17 participants in each group to be able to reject the null hypothesis with a power of 0.80. Expected dropouts are estimated at three participants in each group, which means that the total number of participants needed is 40.

### Statistical analyses

Statistical analyses will be conducted using SPSS (SPSS Inc, Chicago, IL, USA) and SAS (SAS Institute Inc, NC, USA). Data will be anonymized and analyzed by a statistician blinded to the participants’ group affiliation; that is, intervention group or control group. The statistician will remain fully masked during planning and conduction of the statistical analyses. Descriptive statistics for the two groups will be generated. Intention-to-treat analyses with a two-sided significance level at 0.05 will be conducted using the mixed-model repeated-measures method for continuous measures. This model ensures that data values which are missing at random will not create bias. Sensitivity analyses will be used to assess the possible impact of nonrandomly missing values. Prevention of missing values will be attempted by reminding (email and/or telephone) the participants to fill in the questionnaires. Since PEMF therapy is a new approach to treating MCS, per-protocol analyses will be carried out additionally to evaluate a possible treatment effect, if Re5 therapy is applied as specified; that is, compliance ≥80%. The final analyses will be adjusted for baseline levels to improve power. According to the sample size calculation, the two-sample *t* test will have sufficient power to detect a treatment effect, but a mixed model, which utilizes all available data, is likely to be more powerful. The criteria of success in the study are significantly positive changes in the intervention group compared with the control group regarding the primary and secondary outcomes.

### Ethical considerations

The study has obtained approval from the regional ethics committee (registration number: H-1-2013-006) and the Danish Health and Medicines Authority (EUDAMED CIV-ID number 13-01-009621) and is registered with the Danish Data Protection Agency.

In previous studies, only few and mild adverse effects related to transcranial PEMF therapy have been reported. These include mild transient nausea and headache. If serious adverse events should occur, an evaluation will be carefully conducted in order to establish whether the event is related to PEMF therapy or other parts of the study. If an association between the serious adverse event and PEMF therapy is suspected, the allocation sequence for the participant concerned will be unmasked. Any adverse effect or event reported during the study will be registered and reported to the relevant authorities and in future publications.

The study will be conducted in accordance with Good Clinical Practice guidelines and will be monitored by the GCP Unit at Copenhagen University Hospital.

## Discussion

MCS is a condition that may have substantial individual and social consequences. In spite of this, an effective treatment for MCS has not yet been documented. This trial will test whether PEMF therapy can effectively ameliorate the negative effects of MCS in a randomized controlled design. The main strength of the study is that this is the first randomized, double-blind, placebo-controlled trial assessing a possible treatment for MCS. The results will show whether PEMF therapy is beneficial for MCS patients in terms of impact on patients’ lives, symptoms, psychological distress, life quality and whether an associated capsaicin-related CNS response and immunological markers are influenced by the intervention.

Some limitations of the study must be considered. Although the case criteria used in this study are generally accepted, there is no officially approved case definition for MCS and the case criteria are based on self-report because no objective clinical tests are available to confirm the condition.

Furthermore, the study will include MCS patients who experience severe functional impairments because of MCS, which means that the outcomes of the study may not be applicable to all MCS cases. However, in light of the absence of objective clinical tests, functional impairment is a clinically relevant parameter in the assessment of a possible treatment effect. Apart from the fact that a treatment with well-documented effects on MCS is absent, there is very little else to offer these patients in terms of healthcare in general. There is thus a great need for well-conducted randomized trials aimed at assessing possible treatment effects. PEMF therapy is a promising treatment, which can be targeted at the CNS, and a positive outcome of this trial will pave the way for improved healthcare and understanding of this very disabling and overlooked condition.

## Trial status

Participants are being recruited for the study.

## Abbreviations

CNS: Central nervous system; IFN: Interferon; IL: Interleukin; MCS: Multiple chemical sensitivity; PEMF: Pulsed electromagnetic fields; QEESI: Quick Environmental Exposure and Sensitivity Inventory; TNF: Tumor necrosis factor.

## Competing interests

The authors declare that they have no competing interests.

## Authors’ contributions

All authors contributed to the study design. MTDT drafted the manuscript and SS, LA-N, KBC and JE reviewed and revised the manuscript. All authors read and approved the final version.
